# Roberts-SC syndrome, a rare syndrome and cleft palate repair

**DOI:** 10.4103/0970-0358.44939

**Published:** 2008

**Authors:** Jyotsna Murthy, Madhu Dewan, Altaf Hussain

**Affiliations:** Department of Plastic Surgery, Sri Ramachandra University, Porur, Chennai - 600 116, India

**Keywords:** Cleft palate, phocomelia, Roberts-SC syndrome

## Abstract

Roberts SC syndrome is a rare syndrome with only 17 previously recognized patients reported in medical literature. The syndrome is characterized by multiple malformations, particularly, symmetrical limb reduction, craniofacial anomalies such as bilateral cleft lip and palate, micrognathia, and severe growth and mental retardation. Our patient, a young child of five years having Roberts-SC, was successfully operated for cleft palate under general anesthesia. The main features of the syndrome and the technical problems of anesthesia and surgery are discussed in this report.

## INTRODUCTION

Robert-SC syndrome is a rare inherited disorder characterized by nearly symmetrical reductive malformations of the limbs resembling phocomelia. Due to phocomelia-like limb defects similar to those seen in thalidomide embryopathy, the syndrome is also known as the pseudothalidomide syndrome. It is an autosomal recessive disorder with great variability of expression within families first described by Roberts.[1] Later, Herrmann *et al.*[2] described a very similar entity called pseudothalidomide or SC Syndrome. Zergollern and Hitrec[3] concluded that Roberts Syndrome and SC phocomelia are considered the same entity, and therefore, termed it as the Roberts-SC Phocomelia syndrome. These patients have flexion contractures of various joints, micrognathia, scanty hair, delayed growth, microcephaly with or without mental retardation, haemangiomas especially in the head and neck region, corneal opacity, ear anomalies, and may have cleft lip and palate. The typical hand and foot anomalies include an absence of the thumb, shortening of the first metacarpal bone, hypoplasia of the first digit, fusion of the fourth and fifth metacarpals, clinodactyly of the second and fifth digits, and hypoplasia of the middle phalanges. The upper limb is often more severely affected than the lower limb. Other less common features are cryptorchidism, an enlarged phallus (compare to the rest of body), oligohydramnions, renal anomalies (polycystic or dysplastic), heart anomalies (ASD, PDA), and gastrointestinal abnormalities. The unique cytogenetic abnormality called premature centromere separation which disrupts the process of chromatid pairing, is responsible for the development of multiple structural anomalies found in Robert SC Syndrome. Premature centromere separation has been reported in lymphocytes and/or fibroblasts from patients whose clinical phenotypes cover the range of the Roberts syndrome, from a severe variant to SC phocomelia syndrome at the milder end.[4] Jabs *et al.*[5,4] presented evidence that Roberts syndrome is a ‘mitotic mutant.’ They emphasized that, in addition to previously described changes, aneuploidy with random chromosome loss and micronuclei and/or nuclear lobulation in the interphase cells are characteristic features of this syndrome. They suggested however, that the defect might lie in one of the proteins transiently associated with the kinetochore being involved in its function. Stioui *et al.*[6] detected premature centromere separation on chorionic villus sampling at eight weeks' gestation in a woman at a risk of recurrence of Roberts syndrome. Hirschhorn and Kaffe[7] pointed out that they had made a prenatal diagnosis of Roberts-SC syndrome in a family at risk by detection of skeletal and renal abnormalities.[8]

## CASE REPORT

We present here the case of a five year-old boy, born to nonconsanguinous parents. The first child of the parents is seven years old, is normal and was born after nine months of intrauterine life. The second conception resulted in intrauterine death at seven months. Our patient is the third conception and presented with incomplete cleft of the secondary palate. The fourth conception is a one year-old child who is normal.

During the third conception, there was no significant antenatal history of viral fever, medication, or exposure to X-rays(common causes of treatogenicity). The patient was born preterm at 32 weeks by vaginal delivery with a birth weight of 1.8 kg and was kept in the neonatal ICU for seven days. The child had normal head developmental parameters with holding seen at two and a half months and social smiling at three months. There was normal development of the limbs till one year of age after which parents saw very little development in all four limbs. The child was not able to walk or hold anything till two years of age due to deformities of the hands and feet. However, the child had bowel and bladder control by the end of three years.

**Figure 1:**
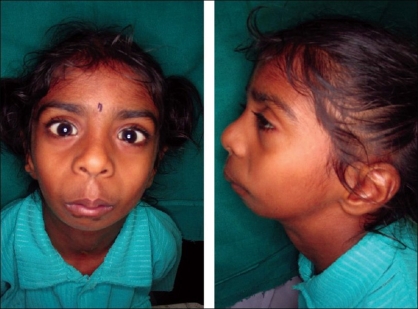
Facial features with retrognathia

**Figure 2:**
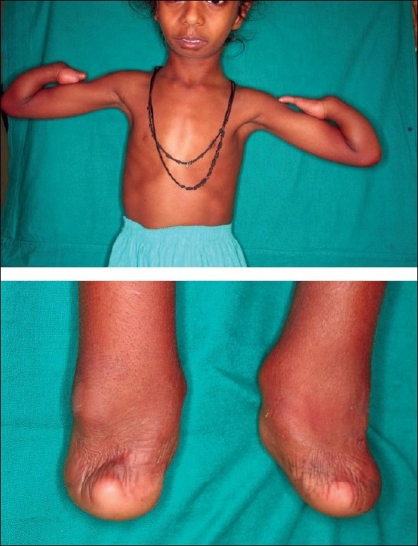
Hand deformities with X-ray

**Figure 3:**
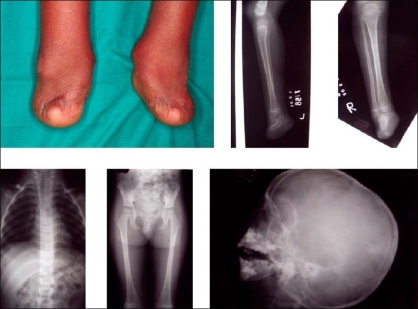
Feet deformities with X-ray of lower limbs, pelvis, clavicles, and skull

The child presented with microcephaly, a bird-like face, and incomplete cleft of the secondary palate [Figure 1]. The weight of the child was 10 kg (expected 14–15 kg), height 91 cm (expected 100 cm), head circumference 43.5 cm (expected 48 cm), lower limb: upper limb ratio 49:52 (expected 1.3:1), and mid-arm circumference 12 cm (expected > 14 cm). An X-ray of the upper limbs showed symmetrical ulnar bone shortening on both sides, the ulna measuring 11.7 cm (expected 13–15 cm) and radii absent from both hands. Only two carpal bones were present in the left and right hands, only one metacarpal on each side and one phalanx on the left side but none on the right [Figure 2]. X-rays of the lower limbs showed the presence of calcaneum only, the rest of the tarsals, metatarsals, and phalanges being absent. The patella was absent bilaterally and there was symmetrical shortening of the tibia by 19 cm (Right) and 19.5 cm (Left) (expected 22.5-24 cm) and of the femur by 20 cm (expected 23–25 cm). An X-ray of the pelvis showed pubic diastasis and a vertically dislocated hip joint. An X-ray of the skull showed micrognathia [Figure 3]. The patient had incomplete cleft of the secondary palate, the cleft being very wide and the soft palate very short. Dental examination revealed a few hypodontic teeth in the oral cavity.

An X-ray of the chest showed malunited fracture of the right clavicle and both clavicles looked rudimentary on clinical examination. An echocardiograph showed S1 (Normal) with S2 wide split but did not show any associated cardiac anomalies. Ultrasonography of the abdomen showed a normal liver (8 cm), normal right and left kidneys, and normal gall bladder, pancreas, and spleen. The haemopoetic system showed microcytic hypochromic anaemia with normal total white cell and normal platelet counts. Liver and thyroid function tests as well as routine karyotyping did not reveal any abnormalities. We could not perform the cytogenetic study due to the lack of specialized markers.

### Surgical and anaesthetic issues

A potentially difficult airway was anticipated in the preanaesthetic evaluation. It was obvious from the facial deformity (retrognathic mandible) that it would render mask ventilation difficult. The mento thyroid distance was adequate (Mallampatti class I) but the choice and size of the endotracheal tube also presented a challenging problem. For his age, the expected size of the endotracheal tube was 5 or 5.5 mm. Following intravenous anesthesia, oral intubation was initially attempted with a 4.5 mm oral endotracheal RAE tube, but as this could not be passed through the glottis, a 4 mm regular endotracheal tube was finally used orally. Intubation was difficult due to retrognathia and an anteriorly placed larynx. The surgical procedure lasted for 60 minutes. There were no significant intraoperative events, and the postoperative anaesthetic course was also without any significant events.

The child's speech assessment was done prior to the operation and it was found that simple monosyllabic sounds and language were delayed. Two years postoperatively, there is a severe language delay as the child is making short sentences albeit only understandable to his family members.

## DISCUSSION

After the first report of Roberts-SC syndrome in 1919 followed by Herrmann *et al.* (1977), approximately 17 cases have been reported. The incidence is sporadic and the recurrence risk is 25% in couples with positive family history. Cytogenetic analysis of fetal cells obtained from chorionic villi samples at the first trimester, amniocentesis, or cord centesis during the second and third trimesters, is required to confirm the diagnosis prenatally. The presence of premature centromere separation clinches the diagnosis. One negative cytogenetic analysis result does not exclude Roberts syndrome. A second analysis using a different type of fetal tissue is required. Termination of pregnancy can be offered when detected before viability, whereas standard obstetrical management in not altered after viability. For those families who have been previously affected, chorionic villi sample for cytogenetic studies must be offered during the first trimester.

Most patients born at term with less than 37 cm of birth length and severe facial and limb defects have been still born or have died early in childhood. Patients born with more than 37 cm of birth length and less severe defects have better prognosis. However, survival beyond infancy is infrequent in patients affected by this syndrome.[9]
